# Building a Discourse-Argument Hybrid System for Vietnamese Why-Question Answering

**DOI:** 10.1155/2021/6550871

**Published:** 2021-12-28

**Authors:** Chinh Trong Nguyen, Dang Tuan Nguyen

**Affiliations:** ^1^University of Information Technology, VNU-HCM, Ho Chi Minh City, Vietnam; ^2^Saigon University, Ho Chi Minh City, Vietnam

## Abstract

Recently, many deep learning models have archived high results in question answering task with overall F_1_ scores above 0.88 on SQuAD datasets. However, many of these models have quite low F_1_ scores on why-questions. These F_1_ scores range from 0.57 to 0.7 on SQuAD v1.1 development set. This means these models are more appropriate to the extraction of answers for factoid questions than for why-questions. Why-questions are asked when explanations are needed. These explanations are possibly arguments or simply subjective opinions. Therefore, we propose an approach to finding the answer for why-question using discourse analysis and natural language inference. In our approach, natural language inference is applied to identify implicit arguments at sentence level. It is also applied in sentence similarity calculation. Discourse analysis is applied to identify the explicit arguments and the opinions at sentence level in documents. The results from these two methods are the answer candidates to be selected as the final answer for each why-question. We also implement a system with our approach. Our system can provide an answer for a why-question and a document as in reading comprehension test. We test our system with a Vietnamese translated test set which contains all why-questions of SQuAD v1.1 development set. The test results show that our system cannot beat a deep learning model in F_1_ score; however, our system can answer more questions (answer rate of 77.0%) than the deep learning model (answer rate of 61.0%).

## 1. Introduction

Question answering is a branch of information retrieval. Many early question answering systems used named entity extraction models to extract answer candidates from the retrieved documents; then, they selected the best five answer candidates for each question. These systems were designed for answering factoid questions; thus, their answers were usually nominal phrases of place, time, person's name, etc. These systems did not answer why-question well because the answers of why-questions are not always nominal phrases. Answering why-questions is a big question for not only many early systems but also recent deep learning models. According to the results of Microsoft Research Asia's R-NET+ (ensemble) model [1], Alibaba iDST NLP's SLQA+ (ensemble) [2], Singapore Management University's Match-LSTM (boundary + ensemble) [3], and Google AI Language's BERT (ensemble) [4] model on SQuAD development set v1.1 published in SQuAD website (https://rajpurkar.github.io/SQuAD-explorer/), we have calculated the why-question F_1_ scores of these models which are shown in Table 1. We can see that the F_1_ scores of why-questions are lower than those of all questions by about 23% in all models. We exploited the SQuAD v1.1 dataset and found that the number of samples with why-question is only about 2700 in training set. This means those models were mostly trained for answering factoid questions.

Why-question answering is an interesting problem. Like how-questions or definition questions, answering why-questions needs a different method from the methods of applying information extraction on information retrieval results. The answers of why-questions usually occur in the form of explanations. The explanations may be arguments or opinions. The important difference between an argument and an opinion is that an argument is either true or false while an opinion is an expression about what a person thinks [5]. Apart from that, many arguments are possibly presented with the same rhetorical structures [6] as opinions. For example, “*The price of book is rising because we have to pay 50$ for it when it was 40$ last week*” is an argument because we can judge whether it is true or false, while “*I love this book because its cover is nice*” is just an opinion and we cannot judge it. According to our surveys, the research on why-question answering is presented in Table 2.

Verberne's why-question answering method is one of the early studies on rhetorical structure approach [7–12]. According to this method, the relevant documents of a why-question are retrieved; then, all text spans which are relevant to the question are selected as answer candidates. These candidates will have additional scores if they are presented in one of six rhetorical structures named Background, Circumstance, Purpose, Result, Cause, and Motivation [13]. In preliminary research on why-question answering [11, 12], Verberne has shown that rhetorical structure of documents plays an important role in answer selection. However, the full rhetorical parses of documents were not easy to obtain; thus, a list of cue words has been used [9, 10] for rhetorical features. The output of this method is a list of passages because it was found that the answer of a why-question may be a passage. Verberne's method has the MRR@150 score of 0.34 with a test set including 187 why-questions.

In the research of why-question answering for Japanese, Higashinaka and Isozaki's method is also a rhetorical structure approach [14]. In this method, Higashinaka and Isozaki use a classifier for identifying which sentence or paragraph has a causal relation to the why-question. Then, the highest-ranking ones are chosen as the final answer. The causal classifier is used because there are many causal structures that do not use any cue word. In other words, a cue word-based feature may miss many causal structures. Therefore, the authors have collected a causal dataset [15] for training a SVM classifier which does not rely on cue words. This method has the MRR@20 score of 0.339 on a Japanese why-question test set. This result cannot compare to Verberne's result because they are not evaluated with the same test set.

The causal classification is also the approach of Oh et al. to why-question answering [16–19]. In early work of Oh et al. [18], the authors solve the problem of causal relation recognition as a sequential labeling problem. They use five tags, namely, B-C, I-C, B-E, I-E, and O, for annotating the beginning of causal part, the inside of causal part, the beginning of effect part, the inside of effect part, and the outside in a text span, respectively. For causal relation recognition, the authors train a CRF (conditional random field) classifier and use it for predicting the causal and effect parts of causal relations. The extracted causal parts are the answer candidates, and they are selected to choose the final answers. This method can find the answers with the precision P@1 score of 41.8% on their developed dataset named WhySet. This result cannot also compare to Higashinaka's and Verberne's results because they use different test sets and evaluation measures. In research on improving why-question answering, Oh et al. also use this causality recognizer to build a large training set for improving the performance of a question-answer classifier [17]. This question answering classifier is used for reranking the answer candidates. In [17], the system using this reranking method has the precision P@1 score of 50% which is higher than that in their previous work on the WhySet dataset. In [19], the authors also use the causality recognizer for extracting causal-effect fragments from 4 billion web pages. These fragments are the references for evaluating the relevance of answer candidates to a why-question. The authors use a multicolumn CNN (convolutional neural network) model called CA-MCNN [19] whose input is a four-tuple containing the why-question, an answer candidate, the causal-effect fragments of the answer candidate, and a reference causal-effect fragment which is the most appropriate to the answer candidate. This method has the precision P@1 score of 54% on the WhySet dataset. The newest work of Oh et al. proposes a GAN-like neural network architecture, which is inspired by generative adversarial nets (GAN) [20], for answer score computation. This network receives a passage and a why-question as input. Then, it generates the compact answer representation of the passage, and the representations of the question and the passage. After that, it computes the answer score of the passage using the representations of the compact answer, the why-question, and the passage [16]. The why-QA system of Oh et al. using this GAN-like neural network has the F_1_ score of 54.8% on the WhySet dataset. When applying this framework to English question answering, the F_1_ scores are from 49.9% to 65.3% and the EM (exact match) scores are from 42.9% to 59.7% on many English datasets including TriviaQA [21]. These datasets contain many question types including why-questions.

The above works show that why-question answering needs a different approach from that of answering factoid questions. The reasonable approach is to select the answers from rhetorical structure parses of answer passages. However, parsing full rhetorical structure of a paragraph or a document is still a big question; thus, these methods focus on recognizing causal-effect relation in the answer passages and use this recognition result as a feature for reranking answer passages. Therefore, we propose our why-question answering method which focuses on five rhetorical relation types, namely, Cause, Result, Purpose, Circumstance, and Motivation [13], and the arguments existing in document for selecting the answers for why-question in Vietnamese. For recognizing the discourse relation of those five types, we analyze the rhetorical structures of answer passages at intersentence level with the five rhetorical relations by using discourse markers and connectives. For recognizing the arguments existing in a document which are not recognized using discourse markers, we use an NLI model to check whether the relation of the two text spans is entailment. For question matching, we also use NLI model with the simple rule that a text matches the question if it implies the question. Our work has three main contributions to why-question answering system. First, we define the answer of why-question using the reason relation concept for explicitly listing the cases where we can find the answer for why-question. Second, we propose a discourse-argument hybrid approach in why-question answering problem to find the answer of why-question as our answer definition. In this novel approach, we analyze the discourse structures of texts with rhetorical structure theory (RST) [6] for identifying the reason parts of the five rhetorical relation types, and we also identify the reason parts by constructing simple arguments in which the contents of the why-questions are the conclusions. Third, we propose a Vietnamese why-question answering model with our approach and implement it with the most appropriate techniques. In this model, we propose a question matching method using an NLI model.

This paper will present our work on building a Vietnamese discourse-argument hybrid system for Vietnamese why-question answering. Our system is the first system integrating both textual argumentation and discourse analysis in identifying the arguments and explanations in a text for answer selection. For building our system, we firstly propose the definition of reason relation and the definition of why-question's answer in reading comprehension context as foundations of answer selection. Then, we apply state-of-the-art models in sequential labeling and natural language inference for solving the problems in argument generation and discourse analysis at intersentential level. Finally, we propose our system architecture for answering Vietnamese why-questions in reading comprehension context. Our contributions are to firstly introduce the why-question answering problem in argumentation and discourse perspective, to propose solutions for the two main problems in this approach, and to finally propose the argumentation-discourse hybrid system for Vietnamese why-question answering in reading comprehension context. Our paper is presented in six sections.  Section 1 introduces our approach in why-question answering and shows the differences between our approach and existing approaches.  Section 2 presents a background on discourse analysis with RST, NLI, and argument generation problems.  Section 3 describes our problem, the approach to solving this problem, and our proposed method for why-question answering.  Section 4 presents our system model for implementing our why-question answering method.  Section 5 describes the datasets and the settings for our system evaluation. Then, some conclusions and future directions are shown in Section 6.

## 2. Background

### 2.1. RST-Style Parsing

**Figure 1:**
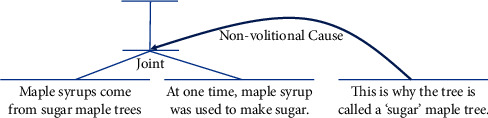
The RST structure of an argument.

Rhetorical structure theory (RST) [13] views documents as sets of rhetorical relations between text units called elementary discourse units (EDUs) [22]. These EDUs are independent clauses. They are nonoverlapping text spans and are not possibly divided into smaller units in documents. The EDUs can combine within certain relations to make larger discourse units, arguments, or opinions [23]. Therefore, RST-style parsing is very important to understand texts at document level. We can identify the premises and the conclusions of an argument or the reasons and the claims of an opinion easily if we have an efficient RST-style parser. Delmonte's example of why-question answering has the RST structure as shown in Figure 1: “*Maple syrups come from sugar maple trees. At one time, maple syrup was used to make sugar. This is why the tree is called a ‘sugar' maple tree.*” This text fragment presents an argument to explain the name “*sugar maple*.” We can easily recognize this argument and identify its premises and the conclusion by exploring its RST structure. This means we can find the answer of why-question in RST structures.

RST-style parsing aims at identifying the document's discourse structure according to rhetorical structure theory [13]. There are two approaches in RST-style parsing. Rule-based parsers [22, 24–26] rely on discourse markers, connectives, and lexicon semantics defined in a verb net or an ontology to identify the discourse parse trees. The rule-based parsers have quite low performances with highest reported F_1_ scores in EDU segmentation and in document level parse of, respectively, 70.35% and 35.44% [26]. Machine-learning-based parsers [27–32] employ sequential labeling and multiclass classification methods for EDU segmentation and discourse relation identification. The performance of machine-learning-based parsers is higher than that of rule-based ones. The highest F_1_ scores of these machine learning parsers are 93.8% [32] in EDU segmentation and 59.9% in document level parse [27]. Although machine leaning parsers have better performance, they have to be trained on a large RST-style discourse treebank which is rare and costly especially in low-resource languages.

### 2.2. Argumentation by Analogy

**Figure 2:**
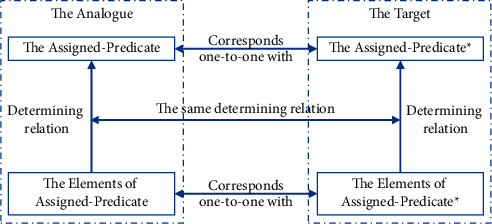
Juthe's argument scheme proposed in [34].

Argumentation aims at studying the argument patterns for generating valid arguments or considering the validity of arguments. People use arguments in all activities in which the analogy arguments are very popular [33]. In research of argument from analogy, Walton et al. [5] have introduced many argument schemes from which a person can make valid arguments; however, these argument schemes are quite difficult to implement in computer programs because each argument scheme is independent guidance which is only understood by humans. Juthe [34] proposes an argument scheme which is possibly applied to make valid arguments.  Figure 2, referenced in [34], illustrates Juthe's argument scheme.

In Juthe's argument scheme, the Assigned-Predicate∗ (the Target) is an argument whose validity should be considered and the Assigned-Predicate (the Analog) is a valid argument. If every element of the Assigned-Predicate has a corresponding element of the Assigned-Predicate∗, and the Assigned-Predicate and the Assigned-Predicate∗ have the same determining relation, then the Assigned-Predicate∗ is a valid argument. In this scheme, an element and its corresponding one must be analogous [34]. This means they must have the same important properties or roles in the arguments. The determining relation is one of many relations, supervenience, causal, truthmaking, correlation, inferential, etc. [34]. Juthe's argument scheme has an important advantage; that is, if we can compute the similarity of two text spans, we might apply this argument scheme for argument validity computation.

### 2.3. BERT Architecture

**Figure 3:**
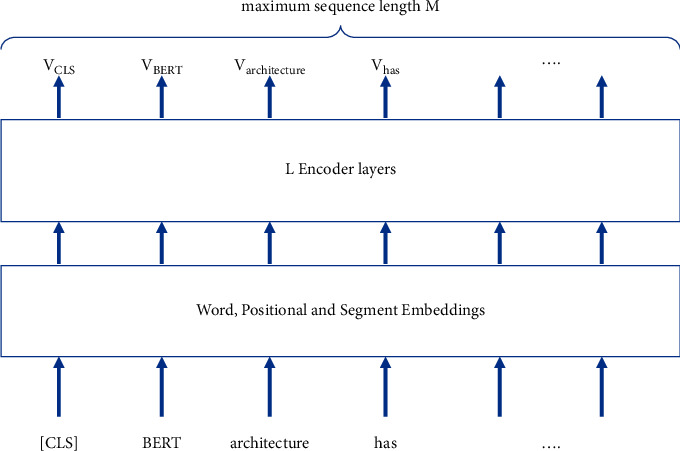
BERT architecture [4].

Bidirectional Encoder Representation from Transformers (BERT) [4] is a multilayer neural network architecture in which each layer is an encoder [35].  Figure 3 illustrates BERT architecture. BERT architecture is used to train neural language models with two tasks: masked language modeling and next sentence prediction. These models, called BERT pretrained models, generate an output vector *V*_token_ for each input token and an output vector *V*_CLS_ for the whole input text. These vectors are calculated from word embeddings, positional embeddings, and segment embeddings of input tokens all at once at each encoder layer. Word embeddings represent the lexicon semantic in distributional semantics. Positional embeddings and segment embeddings represent the effect of a token's position on other tokens' output vectors, so they are possibly considered as syntactic features. Therefore, BERT pretrained model may compute the output vector of each token with both semantic and syntactic features. Many studies [36–38] have shown that BERT architecture computes the context vector of each input token with syntactic and semantic aspects. BERT pretrained models are used in many natural language processing (NLP) downstream tasks by fine-tuning specific training data. The fine-tuned models have shown their state-of-the-art results in many NLP tasks [4].

In BERT models, the input length *M*, the number of encoder layers *L*, the dimension of output vector *H*, and the number of attention heads *A* have significant effect on downstream tasks. These parameters will be selected due to the computation capability in training, fine-tuning, and inference. Devlin's BERT models [4] have two settings. BERT_base_ has the number of input tokens *M* = 512, the number of encoder layers *L* = 12, the dimension of output vector *H* = 768, and the number of attention heads *A* = 12. BERT_large_ has the number of input tokens *M* = 512, the number of encoder layers *L* = 24, the dimension of output vector *H* = 1024, and the number of attention heads *A* = 16. PhoBERT models [39], which are Vietnamese pretrained BERT models, also have two settings as BERT models do; however, PhoBERT models only have number of input tokens *M* = 256, which means we can analyze shorter input text. The performances of these two settings of PhoBERT are slightly different [39]; therefore, we should choose PhoBERT_base_ for fine-tuning downstream NLP tasks in Vietnamese.

BERT pretrained models are used to generate feature vector for each input token; therefore, we need a classifier at the end of BERT architecture for each specific task. The output of each token *V*_word_ or of the whole input *V*_CLS_ will be the input of the classifier. In fine-tuning step, this classifier will be trained jointly with the BERT model with the number of fine-tuning epochs from 2 to 4 to avoid overfitting [4]. Therefore, building an NLP model by fine-tuning a BERT pretrained model is an efficient approach.

## 3. Our Approach

Our approach is to define the answer of a given why-question with a text content by characteristics first. Then, we propose a method of finding the answer in the text content and the model of answering why-question in reading comprehension problem with the necessary techniques for implementing a Vietnamese why-question answering system.

### 3.1. Why-Question Answering with a Single Document

The above why-question answering methods [8, 14, 16–19] have been studied as a task in information retrieval. They find the answers in two phases: passage retrieval and answer ranking. These methods focus on answer ranking which identifies the answer candidates in passages and computes the relevance of these candidates. Recently, many deep models have been proposed for answering questions in SQuAD dataset, where these models have to identify only one answer for a given question and context. The results of these models are shown in SQuAD website (https://rajpurkar.github.io/SQuAD-explorer/). This means the answer candidate extraction has a key role in question answering, and we focus on answer extraction rather than passage retrieval. Therefore, our problem is to find the answer *A* for a given why-question *Q* and context *D*.

Why-questions are raised when people need the reasons. The reasons may be found in arguments or explanations. There is one important difference between an argument and an explanation. According to Johnson and Blair [40], an argument is a claim and the reasons for supporting that claim while an explanation is to provide the information about the origin, cause, meaning, or significance of an event or a phenomenon. When presented in natural language, an argument and an explanation may use similar sentence structures. For example, “*The price of this product is rising because its raw material cost is rising*” is an argument while “*She buys a lot of dresses because it is her preference*” is an explanation. These two sentences are compound sentences linked by the connective “*because*.” This characteristic has been utilized in some research on why-question answering. However, if we build a text classifier by training it on an automatic built dataset for recognizing whether a text span is the answer of a why-question, this classifier may not be efficient because the automatic built dataset may contain both explanations and arguments and these two types are different.

In our approach, we will analyze discourse structure of a document for identifying the arguments and explanations, and we compute the entailment relation of a pair of text spans for identifying the arguments containing one premise and one conclusion. The explanations may be extracted from discourse relations of five types named Cause, Result, Purpose, Motivation, and Circumstance [8, 41]. We use both arguments and explanations in the same way when finding the answer for why-question because they are both used to provide the reasons for an event or a phenomenon. We will find the answer by processing these arguments and explanations.

### 3.2. Definitions

We define the answer *A* of a why-question *Q* = “*Why C?*” given a context *D* for formal answer identification. Our definition about the answer of why-question uses the reason relation concept which is defined as follows.

**Definition 1 .**

(reason relation of two text spans).

Given text spans sp_1_ and sp_2_ in natural language, the reason relation of two text spans sp_1_ and sp_2_, expressed as sp_1_ > sp_2_, is a binary relation defined as follows:

(1) $$\begin{array}{c} {\text{sp}}_{1}⊳{\text{sp}}_{2}\Leftrightarrow \left[\begin{array}{c} {\text{sp}}_{1}≺{\text{sp}}_{2},\\ \text{Cause}\left({\text{sp}}_{2},{\text{sp}}_{1}\right),\\ \text{Result}\left({\text{sp}}_{1},{\text{sp}}_{2}\right),\;\\ \text{Purpose}\left({\text{sp}}_{2},{\text{sp}}_{1}\right),\\ \text{Motivation}\left({\text{sp}}_{2},{\text{sp}}_{1}\right),\\ \text{Circumstance}\left({\text{sp}}_{1},{\text{sp}}_{2}\right). \end{array}\right. \end{array}$$

Here,

- sp_1_≺sp_2_ means sp_1_ is the premise and sp_2_ is the conclusion of an analogy argument
- Cause(sp_2_, sp_1_) means sp_1_ is the satellite and sp_2_ is the nuclei of a Cause relation (Volitional Cause or Nonvolitional Cause) [22]
- Result(sp_1_, sp_2_) means sp_2_ is the satellite and sp_1_ is the nuclei of a Result relation (Volitional Result or Nonvolitional Result) [22]
- Purpose(sp_2_, sp_1_) means sp_1_ is the satellite and sp_2_ is the nuclei of a Purpose relation [22]
- Motivation(sp_2_, sp_1_) means sp_1_ is the satellite and sp_2_ is the nuclei of a Motivation relation [22]
- Circumstance(sp_2_, sp_1_) means sp_1_ is the satellite and sp_2_ is the nuclei of a Circumstance relation [22]

The reason relation defined in Definition 1 has two properties as follows:

- Reflexivity: given text units sp_1_ and sp_2_ in natural language, sp_1_⊳sp_2_
- Transitivity: given text units sp_1_, sp_2_, and sp_3_ in natural language, if sp_1_⊳sp_2_ and sp_2_⊳sp_3_, then sp_1_⊳sp_3_

Intuitively, we can examine whether these two properties are true. For the reflexivity, it is obviously true that everything is the reason of itself, although this does not provide any further valuable information. For transitivity, if sp_1_ is the reason of sp_2_ and sp_2_ is the reason of sp_3_, then we can say that sp_1_ is the deep reason of sp_3_ and thus sp_1_ is the reason of sp_3_ too.

We define the answer of a why-question in Definition 2, which is the foundation for proposing our solution in Vietnamese why-question answering problem. According to this definition, an answer of why-question should be chosen from a discourse structure of a text and the implicit arguments. A discourse structure contains many explanations while arguments in which the content of why-question is the conclusion may not appear in discourse structure. The approaches of Verberne [7–12], Higashinaka and Isozaki [14], and Oh et al. [16–19] try to identify the reason part with a classifier. Because the explanations and arguments are different and the explanations may be explicitly presented in discourse structure while arguments need real world knowledge to be identified, they cannot be identified exactly with one classifier. Therefore, Definition 1 and Definition 2 constitute a novel approach to finding the answer of why-question.

**Definition 2 .**

(the answer of a why-question).

Given a document *D* and a why-question *Q* = “*Why C?*” in natural language, *A* = {sp_1_, sp_2_,…, sp_*k*_} is the answer of question *Q* according to document *D* if all the following conditions are satisfied:

- sp_*i*_ ∈ *D*, sp_*i*_ is a nonoverlapping text span in *D*.
- sp_*i*_⊳*C*.
- ∀*i*, *j* ∈ [1, *k*], *j* ≠ *l*, ${\text{sp}}_{i}\bar{⊳}{\text{sp}}_{j}$. This means two arbitrary text spans of the answer **A** do not make a reason relation. In order words, **A** does not contain any redundant text span.

### 3.3. Finding the Answer for Why-Question

We find the answer of a given why-question and a document with Definition 2. In our approach, we split the document into EDUs for improving F_1_ score because the EDU is the smallest independent clause. Although some why-questions in SQuAD datasets [42, 43] are possibly answered with noun phrases, the answers as clauses are more formal than these phrases. Our answer *A* is a set of EDUs {sp_1_, sp_2_,…, sp_*k*_} satisfying Definition 2.

For identifying the reason relations in document *D*, we will employ a sentence level RST parser to recognize the five discourse relation types described in Definition 1 and an argument generator to generate arguments which contain one premise and one conclusion in document *D*. Our argument generator needs many presuppositions which are valid arguments for entailment recognition. When training or fine-tuning an NLI model, its parameters will be modified to separate the entailment relation from other relations. This means it can encode the valid arguments and compute the analogy of a pair of text spans and the valid arguments. Therefore, we propose using an NLI model for building argument generator.

From reason relations, we can build a directed reason graph in which the vertices are EDUs and the edges are the reason relations of the document. An edge is in the reverse direction of the corresponding reason relation. We will find the answer of question *Q* = “*Why C*?” by identifying the most appropriate EDU, named *S*, for the question *Q*. This means the relation of *S* and *C* is the entailment with the highest score. Then, we find all vertices {sp_*i*_} connected to *S* by breadth-first search. Finally, we select the vertices {sp_*j*_} which do not have any path to other vertices. *A* = {sp_*i*_} is the answer of question *Q* according to Definition 2.

### 3.4. Vietnamese RST-Style Parsing at Intersentence Level

According to the result of many RST parsers, we will not build a full parser at document level, but we will build a restricted RST parser at intersentence level with five discourse relations, Cause, Result, Purpose, Motivation, and Circumstance. In our RST parsing method, we segment a document into EDUs, and then we apply a rule-based parser to recognize those five relations at three levels, named inner-EDU level, inner-sentence level, and intersentence level. At intersentence level, we just recognize the relation between two consecutive sentences. The result of our method is many discourse relations which may not connect to others to form a discourse parse tree because we do not recognize the rest of discourse relations.

#### 3.4.1. EDU Segmentation

We fine-tune a PhoBERT_base_ [39] pretrained model, called UNISeg, for identifying the boundaries of EDUs. First, we create an EDU boundary annotated dataset by exploiting 9046 parse trees from NIIVTB treebanks [44]. We identify all independent clauses in each parse tree and annotate them with a simple rule; that is, all words at the beginning of an independent clause are labeled with “*BC*,” and all remaining words are labeled with “*O*.” With this annotation, an EDU begins with a word labeled “*BC*” and ends at the word before a “*BC*” labeled word or at the last word of the sentence. We use the BERT sequential labeling architecture [4] for fine-tuning PhoBERT_base_ pretrained model on our EDU segmentation dataset. We use the predicted results of UNISeg model to segment a sentence into EDUs with the span based F_1_ score of 0.8. The details of our UNISeg model have been presented in a research article being published.

#### 3.4.2. Intersentence Reason Parser

Our parser recognizes the five discourse relations through inner-EDU, inner-sentence, and intersentence levels and converts them to reason relation according to Definition 1. It identifies the discourse relations at inner-EDU level first; because an EDU is an independent clause, it may include the discourse relations, and if we do not recognize these relations first, they might be wrongly recognized at inner-sentence level. This is also the reason why our method recognizes the discourse relations at inner-sentence level before intersentence level. We build our rule-based parser in 2 phases. The first phase is to identify two context-free grammars (CFG) *G1* = <*Dis*, *N*, Σ, *P1*> and *G2* = <*Dis*, *N*, Σ, *P2*> for inner-sentence and intersentence parsing, respectively. The components of *G1* and *G2* are as follows:

- *Dis* is a primitive symbol which will generate other symbols.
- *N* *=* {*ReasonNS*, *ReasonSN*, *ReasonNN*, *ReasonTM*, *P*, *Word*} is a set of nonterminal symbols. *ReasonNS*, *ReasonSN*, *ReasonNN*, and *ReasonTM* mean the reason relation with nuclei in the left, in the right, and in both the left and the right and the reason relation being recognized, respectively. *P* means a text span including several text spans and discourse markers. Word means a discourse marker.
- Σ is a set of terminal symbols. The terminal symbols are *<span>*, several discourse markers with the form <*discourse-marker*>, and *<punc>* for “,” character.
- *P1* is a set of production rules for inner-sentence parsing.
- *P2* is a set of production rules for intersentence parsing.

The symbol *<span>* in Σ set is the representation of a text span which does not include any “,” characters or discourse markers. This means *<span>* does not contain any discourse relations. Our parser recognizes a string of terminal symbols; thus, an EDU must be converted to string of terminal symbols before passing through the parser. The terminal symbol conversion begins with discourse marker recognition. We recognize discourse markers with the corresponding regular expression patterns. We use a list of discourse markers [45] and specify the recognition pattern for each discourse marker. Then, we split the EDU with discourse markers and “,” characters. Finally, we replace split texts, discourse markers, and “,” characters with *<span>* symbols, corresponding *<discourse-marker>* symbols, and <*punc*> symbols, respectively.

The two sets *P1* and *P2*, which contain context-free production rules, have been built considering text fragments from [45]. These fragments may be sentences or pairs of consecutive sentences. *P1* set contains inner-sentence discourse relation recognition rules which are manually extracted from each sentence. In *P1*'s production rules, the discourse markers may occur at the beginning or in the middle of an EDU or of a sentence. If a discourse relation of the five relations is recognized, we will identify the discourse markers, the nuclei, and the satellite; then, we convert this discourse relation into reason relation according to Definition 1 before adding it to *P1* set. *P2* set contains intersentence discourse relation recognition rules. These rules are extracted from two consecutive sentences using discourse markers. In the five discourse relation types, discourse markers of intersentence relations usually occur at the beginning of the second sentence and rarely occur at the end of the first sentence. We also recognize them and convert them into reason relation according to Definition 1 before adding them to *P2* set. In this building step of grammars *G1* and *G2*, we apply discourse relation patterns which are illustrated in Table 3. Our complete list contains 64 patterns.

For illustration, assume that “*Lý do cho quy tắc số đông là nguy cơ xung đột lợi ích cao và/hoặc tránh quyền lực tuyệt đối*” (in English: “*The reason for the majority rule is the high risk of a conflict of interest and/or the avoidance of absolute powers*”) is a sentence for extracting rules. We consider that this sentence explains the reason of “*quy tắc số đông*” (in English: “*majority rule*”) and the reason is “*nguy cơ xung đột lợi ích cao và/hoặc tránh quyền lực tuyệt đối*” (in English: “*the high risk of a conflict of interest and/or the avoidance of absolute powers*”); thus, “lý do cho” (in English: “*the reason for*”) and “*là*” (in English: “*is*”) are discourse markers. Therefore, we note the pattern “*lý do cho N là S*” with its reason relation and add these rules “*ReasonSN* ⟶ <*lydocho*> *P* <*la*> *P*,” “*Word* ⟶ <*lydocho*>,” and “*Word* ⟶ <*la*>” to *P1*. In these rules, <*lydocho*> and <*la*> stand for discourse markers “*lý do cho*” and “*là*,” respectively. *P2* is built in the same way as *P1*.

The second phase is to propose an algorithm for recognizing intersentence level reason relation from the five discourse relation types.  Algorithm 1 recognizes the reason relations from each EDU with grammar *G1*, then from each sentence with grammar *G1*, and then from multiple sentences with grammar *G2*. In Algorithm 1, each EDU is converted into string of terminal symbols before parsing, and the parsed results are converted into text spans after parsing. In this algorithm, we use function *SentDetect()* for splitting a text into sentences, function *EDUSegment()* for segmenting a sentence to EDUs, function *ConvertToSymbol()* for converting a natural language text to symbols string and a lookup table of pairs of symbols and text spans, function *Earley()* for getting the parse tree containing the highest number of reason relations among many parse trees from a string of symbols, and function *GetRelation()* for getting reason relation from all parse trees.

For evaluation, we use this parser for recognizing the reason relations from 250 text fragments. The results show that it can recognize 78% of reason relations in these 250 text fragments.

### 3.5. Argument Generation

Definition 1 shows that the arguments are also reason relations. Therefore, we employ the NLI solution to make arguments. Our approach is to build an NLI model for verifying if a pair of text spans has a text entailment relation. With this NLI model, we can generate arguments by picking two EDUs *P* and *H*, in which *P* is premise and *H* is hypothesis, and then predict their relation. If the predicted relation is entailment, we have an argument *P*≺*H*. According to Juthe's study in argumentation by analogy [34], if *P* and *H* are analogous to the premise and conclusion of a certain valid argument, then *P*≺*H* is also an argument. Our NLI model may be considered as a function computing the analogy of *P* and *H* with the premises and the conclusions of many valid arguments. These arguments are the entailment samples in training dataset, and the training process also encodes these arguments as the parameters of the NLI model.

We use BERT architecture [4] for building our NLI model because this architecture can compute both syntactic and semantic information of the input text [36–38]. We apply transferred learning approach in building our model. First, we build a Vietnamese NLI dataset, called VSupMNLI, by combining Vietnamese version of MultiNLI dataset [46] with XNLI dataset [47] and our VSupNLI dataset. Our VSupNLI dataset is a Vietnamese native dataset. We combine these two datasets for enriching the Vietnamese version of MultiNLI dataset with Vietnamese native samples from VSupNLI. VSupNLI also provides many samples with which the trained model cannot learn some marks in premises or hypotheses for predicting the relations without computing the semantic similarity of those pairs. Then, we fine-tune PhoBERT_base_ pretrained model on our VSupMNLI and build our model vNLI. Our vNLI model has accuracies of 0.7658 and 0.9665 on Vietnamese XNLI test set and on our Vietnamese VSup test set, respectively.

With vNLI model, we can generate arguments from a document with a simple process. The generated arguments have only one premise and only one conclusion because we can encode a premise and a conclusion as an input text for BERT models only. The argument generating process is presented in Algorithm 2. In this algorithm, we use function *isEntailment()* for verifying if *P*≺*H* is valid with an NLI model.

## 4. Vietnamese Discourse-Argument Hybrid QA System

**Figure 4:**
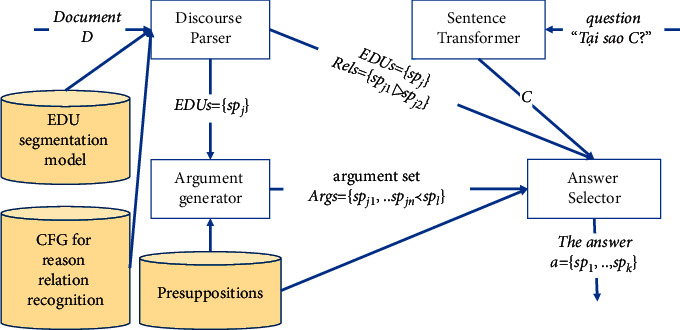
The Vietnamese discourse-argument hybrid QA system model.

We propose our novel Vietnamese discourse-argument hybrid QA system based on our novel approach. Our system is the first system applying discourse analysis and argumentation in solving why-question answering problem. As shown in Figure 4, our system has three key components (discourse parser, argument generator, and answer selector) and one simple component (sentence transformer). Given a document *D* and a question “*Tại sao C*?” (In English: “*Why C*?”), the discourse parser produces a list of EDUs and a list of intersentence reason relations of the document *D* while the sentence transformer converts the interrogative form to affirmative form of the question “*Tại sao C ****?***” Then, the list of EDUs and the list of Rels are passed to the answer selector and the list o EDUs is passed to the argument generator. The argument generator chooses valid arguments in which there are one premise and one conclusion using presuppositions. These arguments are also passed to answer selector. The answer selector builds a reason graph and selects the best answer in the document *D* for the question “*Tại sao C*?” The specific processes of those components are described below.

With vNLI model, we can generate arguments from a document with a simple process. The generated arguments have only one premise and only one conclusion because we can encode a premise and a conclusion as an input text for BERT models only. The argument generating process is presented in Algorithm 2. In this algorithm, we use function *isEntailment()* for verifying if *P*≺*H* is valid with an NLI model.

### 4.1. Discourse Parser

**Figure 5:**
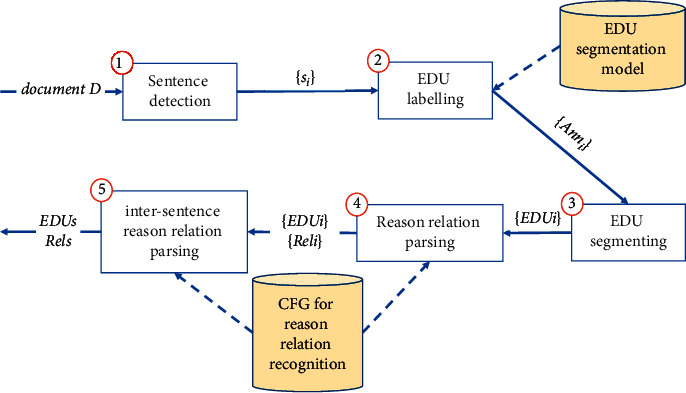
The process of discourse parser component.

The process of discourse parser is presented in Figure 5. The input of this component is the document D. The sentence detection step splits *D* into sentences {*s*_*i*_}. The EDU labeling step, for each sentence *s*_*i*_, predicts the EDU label for all words *Ann*_*i*_ in the sentence using an EDU segmentation model. The EDU segmenting step splits each sentence *s*_*i*_ into EDUs {*EDU*_*i*_} using label predicting results. After that, Each *EDU*_*i*_ of a sentence will be parsed for recognizing all reason relations within each EDU, and then the parsed results of each *EDU*_*i*_ of a sentence will be parsed for recognizing all reason relations within the sentence in relation parsing step, which returns a list of EDUs {*EDU*_*i*_} and a list of reason relations {*Rel*_*i*_} of each sentence. Finally, the parsed results of sentences will be parsed at intersentence level for recognizing intersentence reason relation in intersentence reason relation parsing step. The results of this component are a list of EDUs and a list of reason relations of the document *D*.

### 4.2. Argument Generator

**Figure 6:**
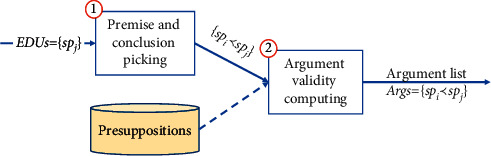
The process of argument generator component.

The process of argument generator, which is the implementation of the Algorithm 2, is presented in Figure 6. The input of this component is a list of EDUs. In the first step, this component picks all pairs of a premise and a conclusion. These pairs may not be arguments; therefore, this component uses presuppositions which are encoded in our vNLI model for computing the arguments' validity in the second step. The result of this component is a list of valid arguments in which there are one premise and one conclusion.

### 4.3. Answer Selector

**Figure 7:**
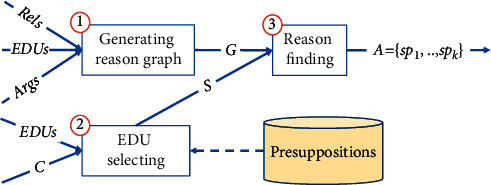
The process of answer selector.

The process of answer selector is presented in Figure 7. In the first step, this component builds a reason graph from an EDU list, an Args list, and a Rels list. The graph's vertices are EDUs of the document *D*, and its directed edges are identified by Args list and Rels list. Each edge has a corresponding argument or relation, where the in-vertex is the premise or the nuclei and the out-vertex is the conclusion or the satellite. In this graph, a tree shows chains of explanations, where the root vertex of the tree is a claim and the leaf vertices of the tree are its reasons according to Definition 2.

In the second step, therefore, it selects an EDU, named *S*, which is the most appropriate to the content *C* of the question *Q*. The appropriate measure of an order pair (*S*, *C*) is the sum of F_1_ score of *S* over *C*, number of nodes in tree *S*, and entailment score of the implication *Sent* ⟶ *C* using presuppositions, which is implemented as vNLI model. *Sent* is the sentence containing *S*. We use entailment score of implication *Sent* ⟶ *C* because the EDU S may not have enough context information; thus, the entailment score of the implication S ⟶ C may be very low although *S* is the most appropriate to *C*. The number of nodes in tree *S* is a heuristic number which is added for choosing the right EDUs because not all EDUs have reason relations in a sentence. A bigger number of reasons means better explanation. The F_1_ score is also added to augment the entailment score. The entailment relation of *Sent* and *C* may have lower score when predicted with vNLI models in practice because vNLI models may not focus on overlapping words which have very different positions in *Sent* and *C*.

In the third step, this component finds the reasons by depth-first search from *S* vertex for identifying the tree with root *S* in the reason graph. Then, all the leaves of *S* tree will be extracted to make the answer *A*. If many EDUs have the same appropriate measure *S* has, this component will identify all the trees and extract all their leaves to make the answer *A*.

## 5. Evaluation

We evaluate our model by implementing a system and testing it as a black box. We use a Vietnamese why-question dataset in which each sample contains a why-question, a context, and an answer for evaluation. Our system predicts the answer of each sample for calculating the F_1_ score. We also compare our results with the results of a sentence retrieval model, of the BERT question answering model, and of a model implemented based on Oh et al. approach [19] to show the advantages and disadvantages of our model.

### 5.1. Datasets

#### 5.1.1. Training Sets

We use a Vietnamese machine translation version of SQuAD v1.1 training set, called viSQuAD, for fine-tuning PhoBERT-YQA model. This training set contains 74,532 samples because we have removed many samples in which the translated answer does not appear in the translated context.

We build a dataset, called VNCE, by extracting causality sentence from Vietnamese news for training a causality recognition model. We use causality patterns defined in regular expressions with many discourse connectives [45], such as “*vì*” or “*bởi_vì*” (in English: “*because*”) and “*để*” (in English: “*for*” or “*in order to*”). We apply these patterns to Vietnamese POS tagged sentences to extract 14,930 sentences. These sentences are automatically tagged with a tag set containing five tags “*B-C*,” “*I-C*,” “*B-E*,” “*I-E*,” and “*O*” as described in Oh et al. [18]. We pick 13,437 annotated sentences for training set and 1,493 annotated sentences for test set.

We also build a training set, called VNANS, for training answer selection model. The VNANS is built with causality sentences of VNCE dataset. Each causality sentence is possibly converted to a why-question and answer pair in which the why-question is the effect part and the answer is the causal part; therefore, we use causality sentences to make positive samples. For creating negative samples, we swap the questions and the answers from positive samples in which the overlapping words of two questions are not nouns or verbs. After creating negative samples, VNANS has a training set containing 13,930 positive samples and 97,510 negative samples and a test set containing 1,000 positive samples and 7,000 negative samples. Thus, we duplicate the positive samples in VNANS training set for balance. As a result, VNANS training set has 208,950 samples.

We use VnCoreNLP [48] for Vietnamese word segmentation and POS tagging when building these above datasets.

#### 5.1.2. Test Sets

We use a Vietnamese human translation version of SQuAD v1.1 development set, called VnYQA, for testing. This test set contains 100 samples which contain only why-questions. We use this translated testing set because the samples are selected by many crowd workers; thus, these samples may be diverse. This set is preprocessed with VnCoreNLP [48] for word segmentation. The statistics of our testing set are shown in Table 4. The test samples may be divided into three groups. In the easy group, the answer of a sample is in a sentence of the context which contains almost the words of the why-question. The answers of easy samples may be easy to identify because we can easily select them using their number of overlapping words with the questions. In the moderate group, the answer of a sample is in a sentence of the context which contains some words of the why-question. With the moderate samples, the TF-IDF scores do not ensure the answer sentence selection because some sentences not containing the answers may have higher TF-IDF scores. In the hard group, the answer of a sample is in a sentence of the context which does not contain any word of the why-question or cannot be identified using our vNLI model and its number of overlapping words with the question. To answer the questions of this group, the model must have some type of inference technique because it cannot rely on word matching. The rates of these groups in our test are shown in Table 5.

### 5.2. Evaluation Settings

#### 5.2.1. VSY-QA Model

We implement sentence retrieval with vector space model, named VSY-QA. For selecting the answer from a context with a why-question (“*Tại sao C*?”), VSY-QA splits the context into sentences and computes the TF-IDF score of each sentence over *C*. Then, it selects the sentence having the highest TF-IDF score.

#### 5.2.2. PhoBERT-YQA Model

We fine-tune a BERT question answering model from PhoBERT_base_ pretrained model [39], named PhoBERT-YQA, using neural network architecture proposed by Devlin et al. [4]. We use Hugging Face library for implementing this task. For answer selection, we select the valid start position and the valid end position where the sum of these positions' scores is the maximum. When predicting the start and end positions with a BERT question answering model, the context is appended after the question to make the input; therefore, the predicted start and end positions may appear in the question span, or the number of tokens between the start and end positions is too big. The valid start and end positions mean these positions are in context span and the number of tokens between them is appropriate. This number is 15 tokens in our setting. We fine-tune PhoBERT-YQA model on viSQuAD with 4 epochs and select the best checkpoint which has F_1_ of 71.26% on Vietnamese version of XSQuAD test set [49].

#### 5.2.3. OH-YQA Model

We implement a why-question answering system, named OH-YQA_causal_, following Oh et al. answer selection method [19] because this method has P@1 of 54% while their latest method [16] has P@1 of 54.8% which is slightly higher than the previous one. In OH-YQA system, we replace the CNN model by our BERT fine-tuned model because a BiLSTM with attention model is better than a CNN model in a text classification task as shown in [50] while a BERT fine-tuned model is better than a BiLSTM with attention model as shown in [4]. We build a causality recognition model by fine-tuning a PhoBERT_base_ pretrained model on VNCE training set and an answer selection model by fine-tuning PhoBERT_base_ pretrained model on VNANS training set. We choose causality recognition model and answer selection model as the best checkpoints when fine-tuning is done with 4 epochs. The causality recognition model has tag-based accuracy of 93.58% on VNCE test set, and the answer selection model has F_1_ score of 78.16% in selecting correct answer.

We also implement a why-question answering system, named OH-YQA_sentence_. This system has only one difference from OH-YQA_causal_; that is, OH-YQA_sentence_ selects the answer from context's sentences; it does not extract the causal part for answer selection.

#### 5.2.4. DA-YQA Model

We build our system, named DA-YQA, following our model described in Section 4. We use Hugging Face library for implementing vNLI and UNISeg models. The vNLI and UNISeg are fine-tuned from PhoBERT_base_ pretrained model with the appropriate architectures proposed by Devlin [4].

#### 5.2.5. Model Fine-Tuning Costs

We use a NVIDIA Tesla M40 12GB GPU to fine-tune all necessary BERT models for our experiment models. The fine-tuning costs are shown in Table 6.

### 5.3. Results

We test the experiment systems on VnYQA dataset with NVIDIA Tesla M40 12GB GPU. The execution time and the GPU memory size of these models are shown in Table 7. The results in Table 7 show that our system needs more resources and it consumes more time than other systems because it uses two BERT fine-tuned models for EDU segmentation and natural language inference, and two stages of RST parsing at inner-sentential and intersentential levels. However, its results in Vietnamese why-question answering are promising.

The test results of the experiment systems are shown in Tables 8 and 9. In Table 8, the answer rate column indicates the number of system's answers containing the gold answer. In general, a system can choose an answer containing more information than the gold answer; thus, its F_1_ score will be low. Therefore, we use answer rate as an additional criterion for comparison. The results in Table 8 show that our system DA-YQA has a better F_1_ score than VS-YQA, OH-YQA_causal_, and OH-YQA_sentence_ systems but it has a lower F_1_ score than PhoBERT-YQA system. However, our system has the best answer rate of 77.0%. This means our system may identify the answer more efficiently than systems PhoBERT-YQA, OH-YQA_causal_, and OH-YQA_sentence_ using other deep neural network models.

**Figure 8:**
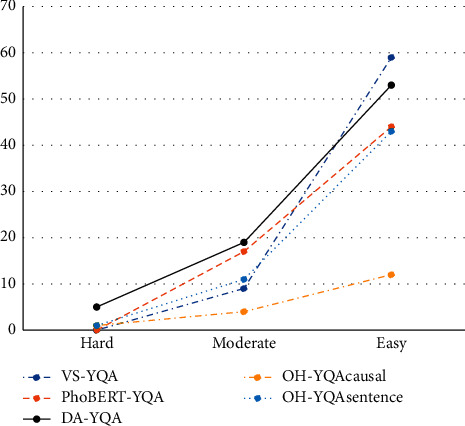
The number of acceptable answers by question groups of VS-YQA, PhoBERT-YQA, DA-YQA, and OH-YQA models.

Table 9 shows the efficiency of our system compared to the four systems VS-YQA, PhoBERT-YQA, OH-YQA_causal_, and OH-YQA_sentence_. We can see these results in Figure 8. Although our system cannot identify all answers in easy samples as VS-YQA system does, it can identify more answers than the four systems in moderate and hard samples. In particular, our system is the best system in identifying the answers in hard samples. These results may indicate that our system has better inference capability than the other four systems. Our system has lower F_1_ score than that of PhoBERT-YQA because our system identifies longer answers than PhoBERT-YQA, and many gold answers are noun phrases while our system's answers are usually clauses. This is also the reason why OH-YQA_causal_ has higher F_1_ score than that of OH-YQA_sentence_. The OH-YQA_causal_ system has lower answer rate than OH-YQA_sentence_ because there are errors in causality recognition which cause wrong result in answer candidate extraction.

The results of OH-YQA_causal_ and OH-YQA_sentence_ systems are the lowest because the answer selection model is not effective with F_1_ score of 78.16% in selecting correct answer. Besides, the method of identifying the causal part in causality sentences needs to be improved because it cannot recognize the causal part in a sentence which contains two nested causal relations. For example, the sentence “*This model is effective because it can run in a low resource configuration thus we apply is in our solution*” has the phrase “*This model is effective*” which is a causal part as well as an effect part. Therefore, the sequential labeling may not be a good choice in causal part extraction. In addition, our training data for answer selection problem is not very large. This is also the reason why our implementations of OH-YQA do not have the expected results.

### 5.4. Discussions

We explore the answers of hard questions from the experiment systems for more details.  Table 10 shows all the hard questions answered by one of the experiment systems and their characteristics to explain the way the systems can find the answers.

According to Table 10, DA-YQA system selects four correct answers from discourse relations and one answer from discourse relations with natural language inference. DA-YQA uses vNLI model for question matching; therefore, it can infer the appropriate sentence of a why-question with related words. Then, DA-YQA selects the discourse related EDU group which is the most appropriate to the question; thus, it can select EDUs in reason relations as the answer. However, the vNLI model is effective in our Vietnamese test set, but it is not effective in XNLI test set or in our Vietnamese why-question answering test; therefore, DA-YQA system does not select correct answers in many cases. The OH-YQA systems do not select correct answers in many cases also because the answer selection model is not effective. Another reason is that OH-YQA systems cannot analyze intersentential discourse relations other than inner-sentential causal-effect relations; therefore, it does not select many correct answers.

## 6. Conclusion and Future Work

In this paper, we would like to present our work on studying a discourse-argument hybrid model for answering a why-question in Vietnamese and implementing a system using this model for evaluation. Our model aims at solving the reading comprehension problem with why-question. For solving this problem, we consider the characteristics of the answers of why-question and then define the answer of the why-question using the concept of reason relation which is also defined in this paper. Our reason relation is a combination of the argument and the five discourse relation types which are used for presenting explanations or arguments. By using reason relations, our model can find 77.0% correct answers while PhoBERT question answering model can find 61.0% correct answers in our test set. This means that our model has better inference capability than PhoBERT question answering fine-tuned model. However, our model has lower F_1_ score (46.49%) because it returns EDU-based answers which are usually longer than the gold answers.

At present, our model can recognize the arguments having one premise and one conclusion, and the intersentence level discourse relations of the five types named Cause, Result, Purpose, Circumstance, and Motivation. These limitations come from the computing limitation of PhoBERT pretrained models which can compute the semantic similarity of two sentences and the lack of large Vietnamese RST discourse bank. However, our model still finds 33.3% of answers from hard samples, which indicates that the approach of combining discourse analysis and argument generation in why-question answering is a promising solution.

At present, our argument generating methods and reason relation parsing are limited at intersentence level; thus, our model cannot find the answer for many moderate and hard samples. In future, we will improve these important methods by researching a model which can compute the validity of arguments containing many premises and many conclusions and researching a discourse parsing model which parses full discourse relations at document level. We believe that these two methods will boost our model's performance significantly.

## Figures and Tables

**Algorithm 1:**
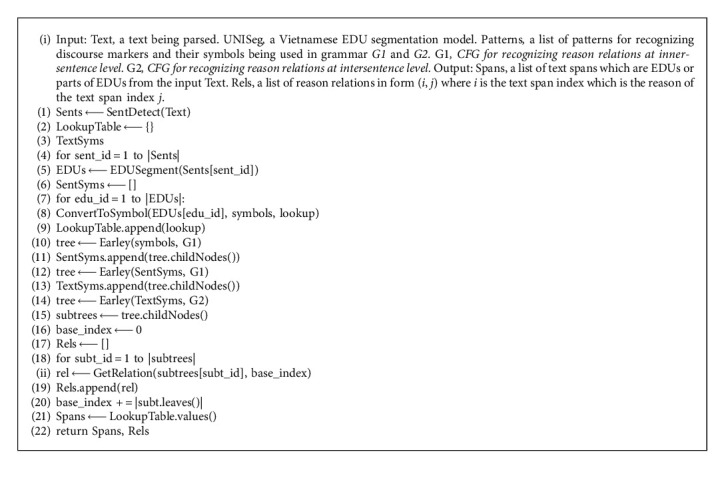
Intersentence reason relation parsing.

**Algorithm 2:**
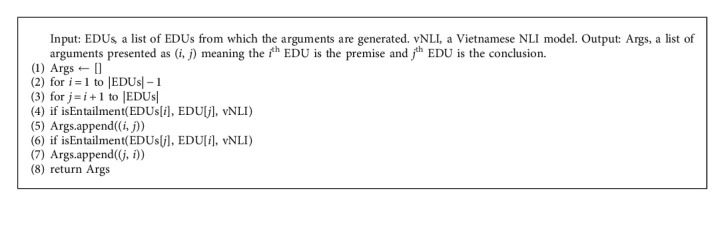
Argument generation.

**Table 1:** The results of some deep learning models on SQuAD development set v1.1.

Model	F_1_	
	All questions	Why-questions
R-NET+ (ensemble)	88.48%	66.90%
SLQA+ (ensemble)	88.38%	65.69%
Match-LSTM (boundary + ensemble)	76.76%	56.95%
BERT (ensemble)	**92.2%**	**69.66%**

**Table 2:** Research works on why-question answering.

Author	Year	Methodology	Dataset	Result
Verberne	2006–2010	IR + RST relation classification	Selected 186 English why-questions on **INEX** corpus	MRR@150 = 0.34
Higashinaka and Isozaki	2008	IR + causal relation classification using SVM	Dataset developed in Japanese	MRR@20 = 0.339
Oh et. al.	2013	IR + causal extraction using CRF	**WhySet**, dataset developed in Japanese	P@1 = 41.8%
	2016	IR + causal extraction using CRF, augmented by adding more training data	**WhySet**	P@1 = 50%
	2017	IR + causal extraction using CRF, answer selection using CNN network	**WhySet**	P@1 = 54%
	2019	IR + GAN-like network (GAN–generative adversarial network)	**WhySet**	P@1 = 54.8%
			**Quasar-T** (https://github.com/bdhingra/quasar)	EM = 43.2% F_1_ = 49.7%
			**SearchQA**	EM = 59.6% F_1_ = 65.3%
			**TriviaQA**	EM = 49.6% F_1_ = 54.8%

**Table 3:** The illustration of discourse relation patterns (*N*: nuclei, *S*: satellite; italics: intersentence relation pattern).

Ord.	Pattern	Pattern meaning	Discourse relation type	Level	Reason relation
1	*S* là nguyên nhân dẫn đến *N*	*S* is the reason of *N*	Cause	Inner-sentence	Reason (S, N)
2	*S. Đây là lý do tại sao N*	***S *** *. This is why * ***N***	*Cause*	*Intersentence*	*Reason (S, N)*
3	*N* với mục đích *S*	*N* with the purpose of *S*	Purpose	Inner-sentence	Reason (S, N)
4	Với mục đích *S*, *N*	For *S*, *N*	Purpose	Inner-sentence	Reason (S, N)
5	*N* phát sinh từ *S*	*N* comes from *S*	Result	Inner-sentence	Reason (S, N)
6	Phát sinh từ *S*, *N*	From *S*, *N*	Result	Inner-sentence	Reason (S, N)
7	*N* nguyên nhân là *S*	*N* because *S*	Cause	Inner-sentence	Reason (S, N)
8	Lý do cho *N* là *S*	The reason for *N* is *S*	Cause	Inner-sentence	Reason (S, N)
9	*N* trong khi *S*	*N* while *S*	Circumstance	Inner-sentence	Reason (N, S) Reason (S, N)
10	Trong khi *S*, *N*	While *S*, *N*	Circumstance	Inner-sentence	Reason (N, S) Reason (S, N)
11	*S. Trong khi đó, N*	*S. Meanwhile, N*	*Circumstance*	*Intersentence*	*Reason (N, S)* *Reason (S, N)*
*…*	*…*	*…*	*…*	*…*	*…*

**Table 4:** Statistics of test set VnYQA.

Criteria	Size (words)
#context	88
#question/answer	100
#context max. length	899
#context avg. length	198
#question max. length	34
#question avg. length	14
#answer max. length	33
#answer avg. length	10

**Table 5:** The rates of easy, moderate, and hard groups in VnYQA.

Groups	#samples	Rate (%)
Hard	15	15.0
Moderate	26	26.0
Easy	59	59.0

**Table 6:** Costs for fine-tuning BERT models used in Why-QA models.

Why-QA model	Costs in fine-tuning time (hour)						
	Answer extraction	EDU segmentation	Causality recognizer	Answer selection	Natural language inference	Total
PhoBERT-YQA	7	—	—	—	—	7
OH-YQA	—	—	1	9	—	10
DA-YQA	—	1	—	—	22	23

**Table 7:** Execution cost of the experiment systems.

	VS-YQA	PhoBERT-YQA	DA-YQA	OH-YQA_causal_	OH-YQA_sentence_
Execution time (seconds per a question)	0.005	0.1	1.93	0.22	0.13
GPU memory size (MB)	—	1.725	2.821	2.273	1.723

**Table 8:** The why-question answering results of the experiment systems.

System	F_1_ (%)	Answer rate (%)
VS-YQA	27.91	68.0
PhoBERT-YQA	52.27	61.0
DA-YQA	46.49	77.0
OH-YQA_causal_	16.95	17.0
OH-YQA_sentence_	23.24	55.0

**Table 9:** The answer rates of the experiment systems.

Models	Hard		Moderate		Easy	
	#samples	Rates (%)	#samples	Rates (%)	#samples	Rates (%)
VS-YQA	0	0.0	9	34.6	59	100.0
PhoBERT-YQA	0	0.0	17	65.4	44	74.6
DA-YQA	5	33.3	19	73.1	53	89.8
OH-YQA_causal_	1	6.7	4	15.4	12	20.3
OH-YQA_sentence_	1	6.7	11	42.3	43	72.9

**Table 10:** The details of the answers from the experiment systems.

Q-ID	Characteristics	DA-YQA	OH-YQA_causal_	OH-YQA_sentence_
9	(i) Circumstance relation at intersentential level	Yes	No	No
12	(i) Circumstance relation at intersentential level	Yes	No	No
44	(i) Inferring related words (ii) Result relation at intersentential level	Yes	No	Yes
67	(i) Inferring related words	No	Yes	No
81	(i) Circumstance relation at intersentential level	Yes	No	No
99	(i) Cause relation at intersentential level	Yes	No	No

## Data Availability

The data used to support the findings of this study have not been made available because they are used in an ongoing study.
